# From Synthesis to Clinical Trial: Novel Bioinductive Calcium Deficient HA/β-TCP Bone Grafting Nanomaterial

**DOI:** 10.3390/nano13121876

**Published:** 2023-06-17

**Authors:** Oleg Mishchenko, Anna Yanovska, Oksana Sulaieva, Roman Moskalenko, Mykola Pernakov, Yevheniia Husak, Viktoriia Korniienko, Volodymyr Deineka, Oleksii Kosinov, Olga Varakuta, Simonas Ramanavicius, Suren Varzhapetjan, Almira Ramanaviciene, Dzanna Krumina, Gundega Knipše, Arunas Ramanavicius, Maksym Pogorielov

**Affiliations:** 1 Department of Surgical And Propaedeutic Dentistry, Zaporizhzhia State Medical and Pharmaceutical University, 26, Prosp. Mayakovskogo, 69035 Zaporizhzhia, Ukraine; mischenko.o.m@zsmu.edu.ua (O.M.); alexeykosinov10@gmail.com (O.K.); varakuta.o.a@zsmu.edu.ua (O.V.); varzhapetyan.s.d@zsmu.edu.ua (S.V.); 2 Theoretical and Applied Chemistry Department, Sumy State University, R-Korsakova Street, 40007 Sumy, Ukraine; a.yanovska@teset.sumdu.edu.ua; 3 Medical Laboratory CSD, Vasylkivska Street, 45, 21000 Kyiv, Ukraine; o.sulaieva@csd.com.ua; 4 Ukrainian-Swedish Centre SUMEYA, Sumy State University, R-Korsakova Street, 40007 Sumy, Ukraine; r.moskalenko@med.sumdu.edu.ua; 5 Department of Morphology, Sumy State University, R-Korsakova Street, 40007 Sumy, Ukraine; m.pernakov@med.sumdu.edu.ua; 6 Biomedical Research Centre, Sumy State University, R-Korsakova Street, 40007 Sumy, Ukraine; yevheniia.husak@polsl.pl (Y.H.); v.kornienko@med.sumdu.edu.ua (V.K.); v.deineka@med.sumdu.edu.ua (V.D.); 7 Faculty of Chemistry, Silesian University of Technology, 44-100 Gliwice, Poland; 8 Institute of Atomic Physics and Spectroscopy, University of Latvia, Jelgavas iela 3, LV-1004 Riga, Latvia; 9 Department of Electrochemical Material Science, State Research Institute Center for Physical Sciences and Technology (FTMC), Sauletekio Av. 3, LT-10257 Vilnius, Lithuania; simonas.ramanavicius@ftmc.lt; 10 NanoTechnas-Center of Nanotechnology and Materials Science, Institute of Chemistry, Faculty of Chemistry and Geosciences, Vilnius University, Naugarduko Str. 24, LT-03225 Vilnius, Lithuania; almira.ramanaviciene@chf.vu.lt; 11 Faculty of Medicine, University of Latvia, Jelgavas iela 3, LV-1004 Riga, Latvia; dzanna.krumina@lu.lv (D.K.); gundega.knipse@lu.lv (G.K.)

**Keywords:** bone grafting nanomaterials, bionanotechnology, maxillary sinus lifting, synthetic bone materials, HA/β-TCP, bionanomaterials, scaffolds, clinical cases, dental implants, nanostructures

## Abstract

Maxillary sinus augmentation is a commonly used procedure for the placement of dental implants. However, the use of natural and synthetic materials in this procedure has resulted in postoperative complications ranging from 12% to 38%. To address this issue, we developed a novel calcium deficient HA/β-TCP bone grafting nanomaterial using a two-step synthesis method with appropriate structural and chemical parameters for sinus lifting applications. We demonstrated that our nanomaterial exhibits high biocompatibility, enhances cell proliferation, and stimulates collagen expression. Furthermore, the degradation of β-TCP in our nanomaterial promotes blood clot formation, which supports cell aggregation and new bone growth. In a clinical trial involving eight cases, we observed the formation of compact bone tissue 8 months after the operation, allowing for the successful installation of dental implants without any early postoperative complications. Our results suggest that our novel bone grafting nanomaterial has the potential to improve the success rate of maxillary sinus augmentation procedures.

## 1. Introduction

Nowadays, the maxillary sinus lift procedure is commonly used to increase the amount of hard tissue in the posterior region of the upper jaw before dental implants are placed. The need for this procedure arises from the phenomenon of sinus pneumatization, which decreases the amount of vertical bone available. The traditional approach to elevating the maxillary sinus involves making an incision in the lateral wall of the maxilla, then lifting the sinus membrane and inserting bone graft under direct visualization [1].

There are several materials that can be used for the maxillary sinus lift procedure, each with its own advantages and disadvantages [2]. Autogenous bone grafts taken from the patient’s own body are considered the gold standard for bone grafting. They have a high success rate and promote natural bone growth. However, they require a second surgical site for harvesting, which can increase the patient’s discomfort and recovery time [3]. Donor allografts, usually from a cadaver, have the advantage of not requiring a second surgical site. They are available in different forms, such as demineralized bone matrix (DBM) or freeze-dried bone allograft (FDBA). However, the success rate of allografts may be lower than that of autogenous grafts, and there is a risk of disease transmission [4]. Xenografts are bone materials derived from a different species, typically bovine or porcine, that are processed to remove all organic matter. They have the advantage of not requiring a second surgical site and are readily available. However, they may not be as effective as autogenous grafts and carry a small risk of an immune reaction [5]. The most successful materials used nowadays for sinus lift procedures are synthetic bone substitutes that mimic the structure and composition of bone. They have the advantage of being readily available and not requiring a second surgical site while also eliminating the risk of disease transmission [6]. However, their success rate may be lower than that of autogenous grafts, and their long-term stability and integration with natural bone may be a concern.

Taking into account the advantages of synthetic bone grafts, different types of such materials are used in clinical practice. Polymers, such as poly(lactic-co-glycolic acid) (PLGA) and polycaprolactone (PCL), are biocompatible and biodegradable and can be designed to have different mechanical and chemical properties [7]. However, their osteoconductivity may be limited, and they may not provide sufficient long-term stability [8]. Bioactive glass, calcium aluminate, and calcium silicate, as bioceramics, are used due to their relatively high biocompatibility and osteoconductivity, as well as being able to promote bone regeneration and angiogenesis [9]. Unfortunately, due to their low mechanical properties, their application is limited [10]. Calcium sulfate-based material, e.g., calcium sulfate hemihydrate (CSH), in addition to biocompatibility and osteoconductivity, can be resorbed and replaced by new bone over time. However, they have a relatively short resorption time and may not provide sufficient long-term stability [11]. The most commonly used materials in clinical practice are calcium phosphate-based materials, including hydroxyapatite (HA) and tricalcium phosphate (TCP), which are both naturally occurring minerals found in bone [12]. Mimicking the natural bone mineral matrix, they are highly osteoconductive and osteoinductive, but HA has a higher crystallinity and slower resorption rate, while TCP has a lower crystallinity and faster resorption rate [13].

In current research, we are investigating novel materials that combine both HA and TCP in one porous graft that can improve graft degradation and osteointegration and provide positive clinical outcomes. The formation of new bone under the influence of transplants is a key goal of sinus lifting and can be achieved through osteogenesis, osteoinduction, and osteoconduction. Osteogenesis occurs in autotransplants by providing a framework, growth factors for osteogenesis, and cells that produce bone matrix. The mechanism of osteoinduction is characteristic of autogenous bone, allogeneic bone, and xenotransplants and involves differentiation of osteogenic cells in response to osteoinductors—bone morphogenetic proteins (BMP) or other growth factors. The mechanism of osteoconduction (osteoconductivity), which involves the use of three-dimensional biological scaffolds, is based on the formation of new bone over the transplant, with the graft potentially undergoing revascularization and incorporation into new bone tissue [14].

The clinical use of any biological materials requires compliance with key requirements for biomaterials and evidence of their safety and effectiveness. Among the mandatory requirements for the use of biological materials from bone tissue are several factors [15]: Firstly, scaffolds must be three-dimensional and have sufficient surface area for interaction with cells and tissues in the area of use. Secondly, the material must be porous and have connections between pores. This requirement is a prerequisite for ensuring cell adhesion, migration, and proliferation of bone cells in the appropriate direction. Effective cell adhesion to the biomaterial and structural anisotropy also affect further cell orientation and cell–matrix interactions. Thirdly, the biomaterial must be non-toxic and biodegradable. However, in doing so, the biomaterial samples must have appropriate mechanical properties, and these characteristics must be comparable to the strength of cortical bone [6].

However, questions of the immunogenicity of biomaterials deserve special attention. Regardless of the specificity of the bone transplant used for augmentation, their implementation is accompanied by an immunological response to foreign substances. It has been shown that, despite careful processing, allogeneic bone retains potential antigenicity, which induces T cell-mediated immune responses against the allograft [16]. This is due to the presence of molecules of the major histocompatibility complex (MHC) in allogeneic bone blocks. It is important to note that the immune system’s response to bone transplants is complex and involves the participation of cells such as T lymphocytes, B lymphocytes, and macrophages.

It is important to emphasize that immune cells provide not only an inflammatory response to foreign material. There is a close relationship between the metabolism of bone tissue and the immune system. Activated T cells can influence bone resorption and osteogenesis through the action of interferon gamma (IF-gamma) or interleukin 17 (IL-17) [17]. RANKL also plays an important role, which binds to the receptor activator of nuclear factor kappa-B (RANK) on osteoclast precursors and induces osteoclastogenesis with subsequent resorption of bone tissue [18].

Under physiological conditions, most macrophages demonstrate the M2 phenotype, which helps maintain tissue homeostasis [19]. Both resident M2 and inflammatory M1 macrophages can affect bone formation. Osteoclasts are traditionally considered resident macrophages in bone. In recent years, a large population of macrophages that constantly reside in bones has been identified. These macrophages are called osteomacs, which can provide pro-anabolic support to osteoblasts and promote bone formation [20]. In the context of implant procedures, macrophages mediate both reparative processes and inflammatory responses to implanted biomaterials [21]. Implants made of bionanomaterials induce the polarization of M1 macrophages, leading to an inflammatory response to foreign bodies and granuloma formation. The mechanisms of the influence of different bionanomaterials on integration, remodeling, and immune response after augmentation procedures are still the subject of active research [22]. Only a small number of studies have provided actual information regarding the tissue response of the periodontal and specific structural composition of the sinus augmentation zone using allogeneic bionanomaterials. Histological evaluation of the bone healing response after the transplantation of different types of bone bionanomaterials in the human body will facilitate the use of bone graft nanomaterial by the surgeon and allow the establishment of the implant healing period according to the patient’s clinical situation. In addition, an assessment of the immune, angiogenic, and osteogenic cell responses to the novel bone grafting material will decipher the mechanisms of the morphogenetic effects of the bone bionanomaterial.

In the current research, we demonstrated a complete circle, from development to clinical application, of novel calcium deficient hydroxyapatite (HA and β-TCP mixture) with a detailed focus on tissue immune reaction and bone remodeling.

## 2. Materials and Methods

### 2.1. Materials

Calcium chloride anhydrous (CaCl_2_), sodium phosphate monobasic anhydrous (NaH_2_PO_4_), and sodium hydroxide (NaOH) were purchased from Pol-Aura (Warszawa, Poland), and sodium bicarbonate (NaHCO_3_) was purchased from Thermo Fisher Scientific (UK). The amount of pure substance was more than 99.5%. Sodium alginate was purchased from Shanghai Macklin Biochemical Technology Co., Ltd. (Shanghai, P.R. China). The reactants were used as received without further purification. All media and chemicals for cell culture experiments and histological evaluation were purchased from Sigma-Aldrich (Darmstadt, Germany) and were used as received.

### 2.2. Bioactive Graft Synthesis

In our study, calcium deficient hydroxyapatite was produced by wet precipitation. The synthesis was carried out under the control of stirring, addition rate, pH, and temperature by the following reaction:

9CaCl_2_ + 5NaH_2_PO_4_ + 13NaOH + NaHCO_3_ → Ca_9_Na(PO_4_)_5_(CO_3_)(OH)_2_ + 18NaCl + 11H_2_O

Two different solutions were prepared separately:

Solution 1.

CaCl_2_ solution (0.09 M) was prepared by dissolving an appropriate amount of CaCl_2_ in distilled water. The solution was heated under stirring up to 80 °C. Sodium bicarbonate (NaHCO_3_) was applied in an amount of 0.01 M as a reactant introducing CO_3_^2−^ groups.

Solution 2.

NaH_2_PO_4_ solution (0.05 M) was prepared. Then, the second solution was added dropwise to the first solution to obtain calcium deficient hydroxyapatite under stirring and heating up to 80 °C for 2 h. During synthesis, the pH of the reaction medium was stabilized at >11 using sodium hydroxide solution. After 2 h, the pH was decreased to 9. The suspension was aged for 24 h at room temperature. The top solution was removed by decantation. The resultant precipitate was washed three times with deionized water until the solution pH became 7, and then it was used as a slurry.

The obtained calcium deficient hydroxyapatite slurry was mixed with 3% sodium alginate water solution in a relation of 3:1. The obtained mixture was added dropwise to the 0.1 M CaCl_2_ solution to obtain granules of HA in an alginate shell. They were frozen at −80 °C overnight followed by drying at 60 °C. The obtained samples were calcined at 900 °C to obtain a mixture of HA and β-TCP and remove the organic phase. The dried samples were ground into fine powders and used for characterization studies.

### 2.3. Bioactive Graft Characterization

The morphology analysis of the obtained hydroxyapatite was performed with scanning electron microscopy (SEO-SEM Inspect S50-B) using an energy dispersive spectrometer AZtecOne with detector X-MaxN20 (Oxford Instruments plc, Abingdon, UK). The X-ray diffraction (XRD) analysis was carried out using an X-ray diffractometer DRON-3M (Bourevestnik, Saint-Petersburg, Russia) connected to a computer-aided system for experimental control and data processing. CuKα radiation was used (wavelength 0.154 nm) with the Bragg–Brentano focusing method. The current and voltage of the X-ray tube were 20 mA and 40 kV, respectively. The scan was performed in a continuous registration mode with a 0.02° step and 1°/min scan speed in a 2 θ range of 20–80°. All experimental data were processed using the DifWin-1 program package (Etalon-TC, Lubertsi, Russia). Phase identification was performed using the JCPDS (Joint Committee on Powder Diffraction Standards) card catalog [23].

The molecule structural components were identified using the Fourier transform infrared spectroscopy method with a ThermoNicolet Nexus 470 apparatus purchased from Thermo Fisher Scientific (Waltham, MA, USA) equipped with an ATR adapter. Measurements and analysis of spectra were carried out with the use of software attached to the device. The spectra were recorded in the spectral range of 550–4000 cm^−1^ with a nominal resolution of 4 cm^−1^ and 32 scans for each measurement. All samples were dried before analysis [23].

### 2.4. Biocompatibility Assessment

Primary osteoblasts (Passage 4) obtained from the collection of the Biomedical Research Center were used to assess the biocompatibility of as-synthesized hydroxyapatite. Before the experiment, cells were cultured in Dulbecco’s modified Eagle’s medium/Nutrient Mixture F-12 (DMEM, Gibco, MA, USA) supplemented with 10% fetal bovine serum, 100 units/mL penicillin, 100 μg/mL streptomycin, and 2.5 μg/mL amphotericin B (Gibco, MA, USA) under conditions of 37 °C and 5% CO_2_. HA powder (100 mg) was placed on the bottom of a 96-well plate, and osteoblasts at a density of 4 × 10^4^ cells/cm^2^ were seeded on over the material. The biocompatibility of the bone grafting material was evaluated using a resazurin reduction assay, as described elsewhere, on days 1, 3, 5, and 7 [24]. Cell proliferation on the tissue culture plate (TCP) was used as a positive control. For that, resazurin was added to the cells at a 15 µg/mL final concentration and incubated for 8 h. One hundred microliters of the medium was then transferred to another 96-well plate, and the optical density (absorbance) was measured using a Multiskan FC plate reader (Thermo Fisher Scientific, Waltham, MA, USA) at 570 and 595 nm. The results were quantified using a formula from the Method for Measuring Cytotoxicity or Proliferation Using Alamar Blue by Spectrophotometry (Bio-Rad Laboratories, Hercules, CA, USA).

Collagen, which was synthesized by osteoblast cells and accumulated on samples, was detected through staining with Sirius Red dye. The staining was performed as follows [25]: Cells were seeded on over the material at a cell density of 10⁴ cells per well, and on the 7th and 14th days of incubation, the samples were transferred to another 24-well plate and washed 3 times with ice-cold PBS (40 °C). Then, 1.5 mL of Bouin’s solution was added to each well for 1 h at room temperature. After the solution was removed, the samples were rinsed with cold tap water and dried in a fume hood overnight. On the next day, 1.5 mL of Sirius Red dye was added to the samples for 1 h, then removed, and each well was washed 4 times with 0.01 M HCl. NaOH solution (1 mL of 0.1 M) was added to each well in order to recover the bound dye. The plate was placed on a shaker for 30 min, after which 100 µL of eluted dye from each well was transferred to a 96-well plate, and the absorbance was measured using a Multiskan FC (Thermo Fisher Scientific, Waltham, MA, USA) plate reader at a wavelength of 570 nm.

### 2.5. Blood Interaction Test

A total of 4.0 g of bone grafting nanomaterial was weighed and placed in six Petri dishes with a diameter of 6 cm. In order to control the speed of blood clotting, six additional empty Petri dishes were prepared. Whole venous blood (60 mL) was collected from a male volunteer who provided consent for the study. Prior to conducting the study, the Ethics Committee on Medical Research of the Medical Institute of Sumy State University approved the protocol. Next, 5 mL of blood was immediately added to each dish containing the bone grafting nanomaterial. The samples were gently stirred with a glass rod to ensure even distribution of the blood. A timer was started as soon as the blood was added, and it was stopped once a clot had formed. For scanning electron microscopy (SEM) analysis, bone grafting nanomaterial with coagulated blood weighing approximately 0.5 g was fixed in a 2.5% glutaraldehyde solution and then dehydrated in alcohols of increasing concentration for 24 h. Once dry, the samples were covered with a 30–50 nm layer of silver using a vacuum set-up VUP-5M (SELMI, Sumy, Ukraine). The SEM images of the blood clot on the hydroxyapatite were captured using an FEI Inspect S50B (FEI, Brno, Czech Republic) with an Everhart–Thornley secondary electron detector.

### 2.6. Animal Experiment

**Figure 1:**
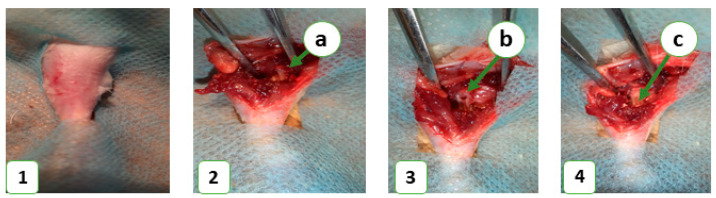
Operative procedure for bone defect plastic. 1—prepared area before the operation; 2—general view of the operative area (**a**)—bone before defect formation; 3—general view of bone defect formation stage (**b**)—bone defect; and 4—general view of the last operation stage (**c**)—defect filled with bone grafting nanomaterial.

In this study, 36 laboratory rats were obtained from the Vivarium of Sumy State University. The animals were housed at 22 ± 2 °C on a 12-h light/dark cycle and had free access to food and water. Each animal was kept in a separate cage in accordance with the Directive 2010/63/EU of the European Parliament and of the Council of 22 September 2010 on the Protection of Animals Used for Scientific Purposes. The study was approved by the Commission on Bioethics Compliance in Experimental and Clinical Research. The animals were randomized into control and experimental groups, with 18 animals in each. The control group consisted of animals that did not receive any bone substitute after the operation, while the experimental group included animals that received a novel bioactive bone graft nanomaterial to replace a bone defect in the middle third of the tibia. Prior to the operation, the animals’ legs were shaved under anesthesia (ketamine, 10 mg per 1 kg). The surgical field was treated with 70% ethanol to prevent bacterial contamination and then surrounded with a sterile cloth. A bone defect was created in the middle third of the tibia using a stomatological drill (d-2.2 mm) and filled with the novel bone grafting nanomaterial in the experimental group (Figure 1). The wound was closed with simple interrupted sutures, and an aseptic dressing was applied. The animals were euthanized on the 7th, 14th, and 28th days of the experiment with an overdose of ketamine (70 mg/kg).

The material was fixed in a 10% neutral (buffered) formaldehyde solution for 24 h. All tissue processing procedures (fixation, decalcification, paraffin saturation, and embedding) were performed according to generally accepted methods. Serial sections with a thickness of 4–5 μm were stained with Mayer’s hematoxylin and eosin.

### 2.7. Clinical Application

**Figure 2:**
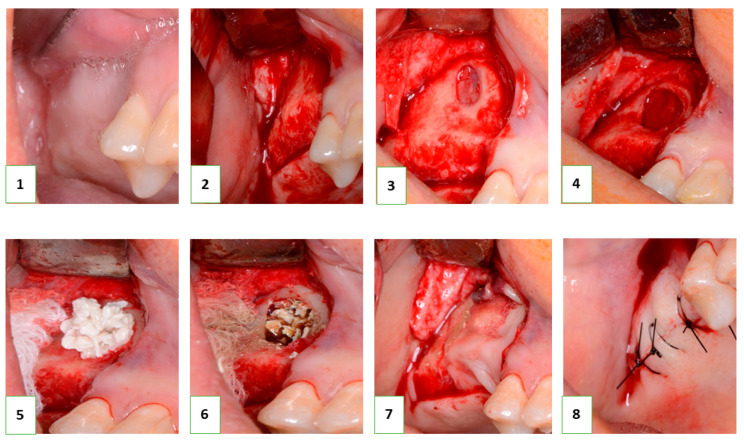
Sinus lift procedure (description is in the text). **1**—Right maxilla, a defect in the dental arch; **2**—The trapezoidal mucosal–periosteal flap has been formed and detached, and the alveolar process and anterior wall of the right maxillary sinus have been skeletonized; **3**—A window has been formed in the anterior wall of the right maxillary sinus; **4**—Mobilization of the Schneiderian membrane to the height of the planned augmentation; **5**—Loading, distribution, and condensation of the graft into the area of the lower wall of the right maxillary sinus; **6**—Control of the degree of vascularization of the recipient site and control of the integrity of the Schneiderian membrane; **7**—Introduction of the APRF membrane under the mucosal–periosteal flap in the area of the window on the anterior wall of the maxillary sinus; **8**—The mucosal–periosteal flap is placed back in its original position and the wound is tightly sutured.

Following the successful in vitro biocompatibility assessment and in vivo evaluation of effectiveness, the bone grafting nanomaterial was approved for clinical application under protocol #12-75/19 (Zaporizhzhia State Medical University). The open sinus lifting operation was carried out according to the following protocol (Figure 2): After sedation with an analgesic (Ketanov) and application of a hemostatic agent (Dicinon), a full-thickness mucoperiosteal flap was formed in the area of the anterior wall of the maxillary sinus, followed by skeletonization of the anterior wall of the maxillary sinus. Using a round bur with irrigation, the cortical layer of the bone was removed to expose the sinus membrane (Schneider’s membrane). The membrane was then peeled from the floor of the maxillary sinus and the side wall of the nose using sinus elevators and raised to the height of the desired augmentation. The space formed between the bottom of the sinus and the dome of the membrane was filled with a graft that had been pre-moistened with an antibiotic and antiseptic solution (dioxidine, chlorhexidine bigluconate 0.05%). The graft was evenly distributed throughout the volume and condensed with a force of up to 150 g/cm^2^. The integrity of the Schneiderian membrane was monitored, as well as an assessment of the degree of vascularization of the recipient zone by the rate of wetting of the augmentate with blood. The window in the anterior wall of the maxillary sinus was closed with a membrane (PLA), the mucoperiosteal flap was placed in place and sutured, and standard anti-inflammatory therapy was prescribed. The sutures were removed on the tenth day, and no complications were observed in the postoperative period.

In all cases, implantation was recommended to patients after 8 months. In the next stage, the installation of screw implants was carried out according to the standard protocol, with simultaneous sampling of a bone fragment from the augmentation area using a tubular burr. The terms of implantation varied from 8 to 12 months and were determined based on the clinical situation. This clinical study included 6 clinical cases with a detailed analysis of tissue biopsy before implantation.

### 2.8. Histological Evaluation of Bone Augmentation with HA/β-TCP

Intervention and further biopsy were performed in line with the Declaration of Helsinki. The study protocol was approved by the ethics committee (protocol #12-75/19, Zaporizhzhia State Medical University). All patients gave their informed consent before enrollment in the study, and all patients completed the study successfully and received the opportunity for free follow-up visits after the intervention.

The samples obtained after biopsy were fixed immediately by immersion in 10% buffered formalin for 24–48 h with further decalcification in EDTA (4.1% disodium ethylenediaminetetraacetic acid solution). After completion of decalcification, the biopsies were processed according to the standard protocol with further embedding in paraffin (Paraplast). Paraffin blocks were cut at 4–5 µm, and histological slides were stained using hematoxylin and eosin, as well as toluidine blue, for routine histological examination. Examination of histological specimens was performed using parameters adapted from those used for the assessment of bone healing and remodeling. The shares of space filled with bone trabeculae, graft nanomaterial, and connective tissue were measured histomorphometrically [26]. In addition, the thickness of the bone trabeculae was measured in three areas of three serial sections at three representative areas at high magnification (Leica Microsystems GmbH, Wetzlar, Germany). The intensity of osteogenesis was assessed semi-qualitatively using the following scoring system: 0 =  no features of osteogenesis; 1 = bone formation around graft (e.g., granules) with the appearance of osteoblasts and deposition of osteoid; 2 = bone formation around graft with primary bone features; 3 = bone trabeculae formation around grafts with features of active remodeling (detection of lamellar bone with primary osteons and osteocytes, vascular detection, fibrous bone remnants in the lamellar bone, and remains of bone replacement nanomaterial embedded in/on the bone); 4 =  mature lamellar bone with osteons [14]. In addition, the immune reaction to NG was assessed using a semi-quantitative score according to the following scheme: 0 = none; 1 = loose infiltrates, disseminated or focal; 2 = dense, moderately extensive lymphocytic infiltrates; 3 = extensive, dense lymphocytic infiltrates with edema and focal giant cells; 4 = pronounced inflammatory reaction including giant cells and necrosis [27]. The assessment was performed by two independent observers blindly

### 2.9. Immunohistochemical Study

To analyze the host tissues’ and cells’ reactions to NanoGraft, an immunohistochemical (IHC) study was performed. Serial sections 4 μm in thickness were cut, deparaffinized, and hydrated. Endogenous peroxidase activity was blocked using 3% methanol in hydrogen peroxide. After antigen retrieval, incubation with primary antibodies was performed. After washing, labeled polymer secondary antibodies (Envision Detection System, Dako) were added to the slides. Peroxidase activity was detected using diaminobenzidine (DAB), yielding a brown staining product. Slides were counterstained with Mayer’s hematoxylin. The following biomarkers were used for IHC: CD8 (DAKO; Clone C8/144B) was used for visualizing cytotoxic T cells as effector cells of cell-mediated immunity, and FOXP3 (Cell Marque, Clone EP340) was used for the assessment of T regulatory lymphocytes producing anti-inflammatory and profibrogenic agents. CD68 (DAKO, Clone KP1) and CD163 (Cell Marque, Clone MRQ-26) were used to assess macrophages of the M1 and M2 types. For evaluating angiogenesis, we used antibodies against CD34 (DAKO, Clone QBEnd 10). Finally, to analyze the osteogenic potential of the graft, SATB2 (Cell Marque, Clone EP281) as a marker of osteogenic lineage differentiation was applied.

To evaluate the effect of augmentation on the overall outcome, histomorphometry was performed with an evaluation of the proportion of the specimen filled with bone tissues, graft nanomaterial, and connective tissue. For assessing the potential immunogenic effect of the bionanomaterial used for augmentation, the intensity of inflammation was examined using semi-quantitative analysis. In addition, the presence and density of innate and adaptive immunity cells were considered adaptive. Reactions of pro- (CD68+) and anti-inflammatory (CD163+) subtypes of macrophages were assessed. In addition, the density of immunoreactive (CD8+) and immunosuppressive (FOXP3+) T cells was scored. To consider angiogenesis, the number of vessels was evaluated using cells positive for vascular biomarker CD34. The characteristics of the biomarkers are presented in Table 1.

To make a judgment about the osteogenic properties of the graft, we assessed bone tissue formation by measuring the proportion of bone trabeculae in the specimen and the density and spatial distribution of osteogenic cells highlighted by SATB2. Digital photographs of the histological and immunohistochemical specimens were taken using a digital camera (Leica Microsystems) placed over a light microscope (Leica Microsystems).

### 2.10. Statistical Analysis

Quantitative data were expressed as mean ± standard deviation. Comparisons between groups were performed using the *t*-test. In addition, the Kruskal–Wallis test was used when working with categorical data. A *p* < 0.05 was determined to be statistically significant. Statistical analyses were performed using GraphPad Prism 8.0 (V8.0.1).

## 3. Results

### 3.1. Electrodeposition of Molecularly Imprinted Polypyrrole

**Figure 3:**
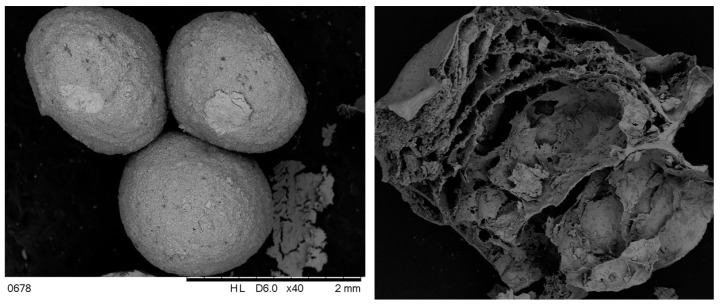
Scanning electron microscopy of HA/β-TCP bioactive nanomaterial (**left**, Magnification ×40) with cross-section image (**right**, Magnification ×100).

Novel HA/β-TCP is a nanomaterial represented by granules with an average size of 1.4 ± 0.30 mm in diameter with interconnected pores. The pore size varied from 25 ± 3.8 µm to 196 ± 25.6 µm in the lateral dimension (Figure 3). The pores are made due to the elimination of the organic phase during the cycle of freezing and heating (from −80 °C to 900 °C). The formation of pores will allow for blood and cell penetration, which is critical for osteoconductivity and osteointegration [28]. Taking into account the different degradation behaviors of HA and TCP, the last one will be removed from the graft within a short period of time (approximately 2–3 weeks) [29] and will be substituted with newly formed bone tissue. This feature of novel HA/β-TCP will keep a balance between nanomaterial resorption and bone tissue ingrowth.

**Figure 4:**
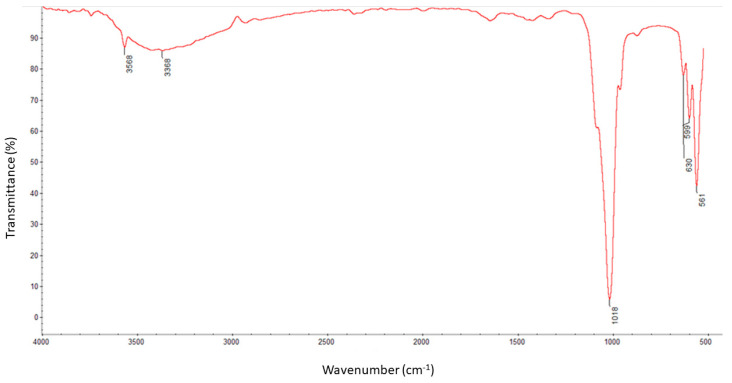
FTIR analysis of bioactive bone graft.

Figure 4 demonstrates the FT-IR spectra of HA/β-TCP bioactive nanomaterial. The absorption peaks located at 1018 cm^−1^ originated from asymmetrical stretching (ν3) of PO_4_^3−^, and at 561 and 599 cm^−1^ were attributed to bending modes (ν4) of PO_4_^3−^, respectively. The symmetric stretching modes (ν1 and ν2) of PO_4_^3−^ were also observed at approximately 961 cm^−1^, while a weak sharp peak at 3568 cm^−1^ corresponded to the stretching vibration of the lattice OH- ions [30]. The typical bands of HA that can be assigned to the PO_4_^3−^ asymmetrical stretching located at the vibrational frequency of 1018 cm^−1^ (ν3), 599–561 cm^−1^ (ν4), and O-H stretching vibration at 3368 cm^−1^, were found in an obtained sample of hydroxyapatite. Bands at approximately 1400–1415 cm^−1^ (ν3) and 870 cm^−1^ (ν2) can also be observed due to the presence of CO_3_^2−^ [31].

**Figure 5:**
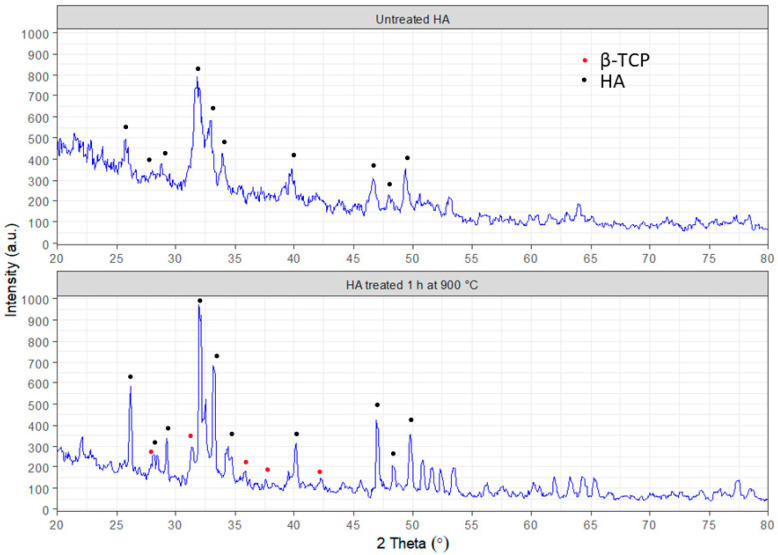
XRD of synthesized calcium deficient hydroxyapatite as prepared dried powder (**upper** image) and sintered powder at 900 °C (**lower** image).

The results of XRD analysis (Figure 5) show that the prepared sample corresponds to hydroxyapatite (JCPDS 9-0432). HA can be termed non-stoichiometric due to the slight shift of main peaks to the right. After treatment of samples at 900 °C, the additional phase of β-TCP is observed. It corresponds to the card (JCPDS 09-169) [4]. The diffraction patterns obtained for the as-prepared sample confirm the presence of a poorly crystalline apatite phase with no other extra peaks for the powder. However, calcination at 900 °C affected the formation of the β-TCP phase for the sintered sample, which could be attributed to the transformation of calcium deficient apatite to biphasic mixtures of HA and β-TCP, as evident from Figure 4. The substituted monovalent ions, including Na^+^ for Ca^2+^, in the apatite structure cause a charge imbalance that can be neutralized by creating supplementary vacancies [32] or by the occurrence of simultaneous substitutions of cations and anions, such as in the case of substitution of Ca^2+^ by Na^+^ and of PO_4_^3−^ by CO_3_^2^, without any vacancy creation or loss of charge balance [32,33].

### 3.2. Blood Interaction Test, Biocompatibility, and Animal Experiment Results

**Figure 6:**
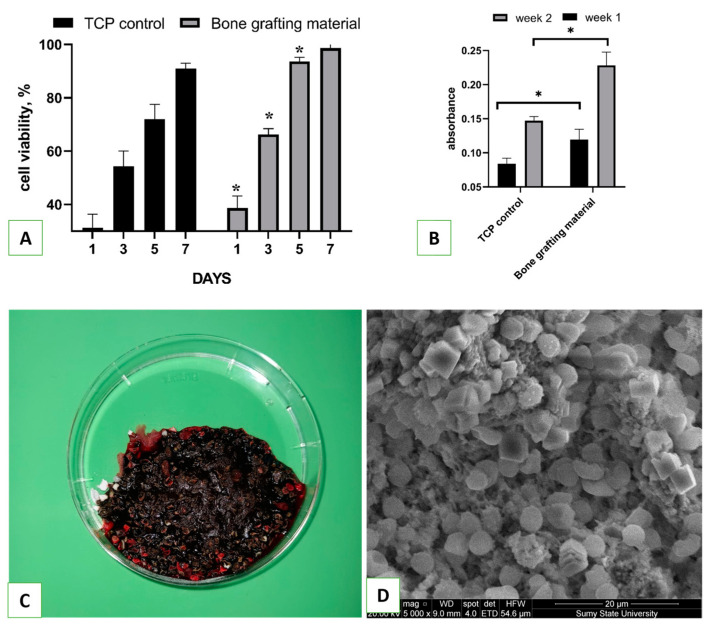
Human osteoblast cell viability assay during 7 days of cultivation with HA/β-TCP bioactive nanomaterial (**A**) with the collagen production assay in weeks 1 and 2 after cell seeding (**B**). *—statistical significance (*p* ≤ 0.05 between control end experimental groups). Image of blood clot formation within one minute after the material interacted with human blood (**C**) with SEM image of HA/β-TCP bioactive nanomaterial (**D**) after the blood interaction experiment.

Osteoblast cells demonstrated osteoinductive patterns of the novel HA/β-TCP bioactive nanomaterial: The cell viability assay showed significantly better cell attachment on day 1, as well as osteoblast proliferation on days 3 and 5, compared to the TCP control (Figure 6A). Inorganic calcium phosphate is known as a natural stimulator of bone cell proliferation and differentiation [34] and, due to β-TCP, the novel nanomaterial exhibited advanced osteoinductive properties. In addition to cell proliferation, the novel nanomaterial demonstrated stimulation of collagen expression—the level of collagen in weeks 1 and 2 increased two-fold compared to the TCP control. In previous research, we demonstrated that HA could be a specific factor for the stimulation of collagen expression, but here, the combination of both HA and β-TCP provided a significant advantage for novel bioactive nanomaterials as bone substitute graft.

Just after the biomaterial application during the sinus lift procedure, it contacts with blood, which is an initial phase of tissue organization. The blood interaction experiment demonstrated that the average time for blood clot formation in contact with hydroxyapatite was 55 ± 17 seconds. While the nanomaterial was hydrophilic, it did not absorb the entire volume of blood without some mixing. However, once formed, the clot strongly adhered to individual fragments of the samples as well as the bottom of the dish (Figure 6C). In contrast, whole blood in Petri dishes without bone grafting material clotted in 3.4 ± 22 minutes. Interestingly, when whole venous blood was introduced to hydroxyapatite, there was a rapid color change from dark red to bright red with a green tint, indicating an interaction between the blood and the material. In contrast, blood without any samples remained dark red. SEM demonstrated that the HA/β-TCP bioactive nanomaterial was covered by blood cells, mainly platelets and erythrocytes, with the formation of thin fibrin fibers (Figure 6D). It is known that Ca ions can stimulate ADP-induced aggregation of human platelets and facilitate fibrinogen transformation to fibrin [35]. In our experiment, we can see the formation of a blood clot after interaction with blood, which made a “natural organic scaffold” around the bioactive bone graft that could stimulate the formation of new bone after material placement to the maxillary sinus.

**Figure 7:**
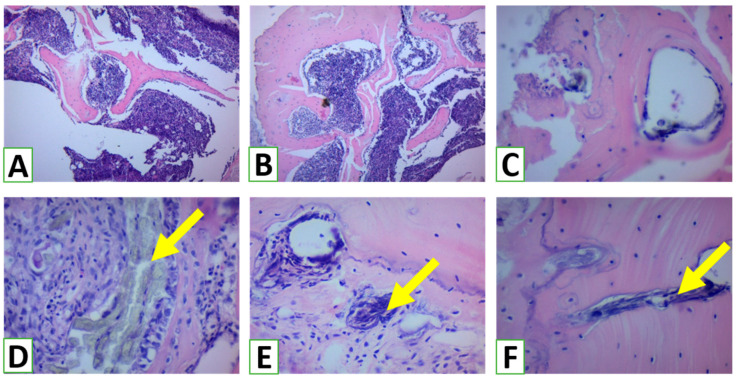
Histological evaluation of bone defect zone after bone trauma on days 7, 14, and 28 in the control group (**A**–**C**) and after application of HA/β-TCP bioactive material (**D**–**F**). Arrows demonstrate remnants of HA/β-TCP nanomaterial. Hematoxylin and eosin staining. Magnification ×100.

The formation of new bone in the animal experiment passed through the formation of the hematoma and inflammatory stages with granulation (day 7), the beginning of new bone formation (day 14), the formation of mature bone (day 28), and remodeling (not performed in our experiment). Both the control and experimental groups demonstrated normal osteogenesis, but the HA/β-TCP group had some important differences (Figure 7). The remnant of the bone grafting materials on the 7th day allowed for compact granular tissue formation with more rapid bone ingrowth on day 14. On the second time point, we observed more rapid calcification, probably due to the additional source of inorganic Ca and P from the β-TCP phase. On day 28, the newly formed bone in the HA/β-TCP group demonstrated a more compact structure and the formation of mature osteons. The remnant of bioactive materials was still present, demonstrating low bioresorption of the HA phase in the novel nanomaterial.

### 3.3. Clinical Outcomes

**Figure 8:**
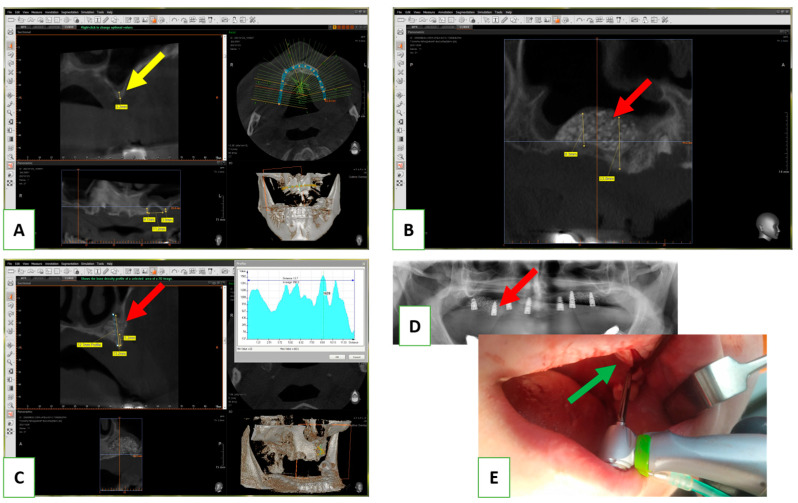
CT scans of a patient with complete edentulism of the upper jaw and a deficiency in bone tissue volume in the lateral sections of the upper jaw before sinus lifting (**A**); 6 months after the procedure (**B**,**C**); and after the dental implant operation (**D**) with bone sample harvesting (**E**). Yellow arrow—bone deficiency site; red arrow—HA/β-TCP bioactive nanomaterial; green arrow—place of bone sampling.

After the sinus lifting procedure, there were no significant complications in any patient during the 8–12 months of observation. With the aim of bone regeneration, control CT scans after 6 months were performed for all patients, and bone quality control was performed. We observed the formation of good quality bone that would allow for dental implant placement in the period from 8 months to 1 year. Figure 8 (representative case) demonstrates the CT scan of a patient with complete edentulism of the upper jaw and a deficiency in bone tissue volume in the lateral sections of the upper jaw (Figure 8A). In 6 months after sinus lifting with the novel HA/β-TCP bioactive nanomaterial, we observed the formation of novel bone tissue inside the material (Figure 8B,C). The implantation procedure performed 9 months after sinus lifting with the novel nanomaterial (Figure 8D) showed no clinical complications in the early postoperative period.

**Figure 9:**
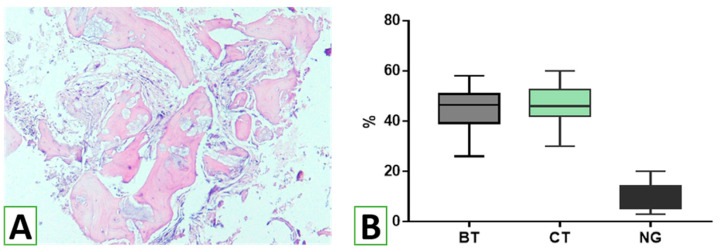
Structural components of the sinus augmentation zones. Osteogenesis features at the place of sinus augmentation are visible (**A**). Numerous bone trabeculae (BT) of variable thicknesses and structures are found around the remnants of HA/β-TCP (NG). The spaces between bone trabeculae are filled with connective tissue (CT). The structural assessment of the tissue from the biopsy (**B**) revealed equal volumes of bone trabeculae (BT) and connective tissues (CT) and small remnants of HA/β-TCP (NG). (**A**)—histological specimen of the biopsy material from the zones of augmentation. Staining with hematoxylin and eosin. Magnification ×40.

Before implantation, a bone biopsy was performed after the informed concern. The assessment of tissue biopsies showed histological features of osteogenesis with signs of active bone remodeling. Considerable areas of the biopsies were filled with branched bone trabeculae of variable thicknesses, ranging from 20 to 190 µm. Histomorphometry revealed that bone trabeculae possessed 44.6 ± 1.73% (95% CI 41.1–48.2%) of the biopsy tissue volumes (Figure 9). At the same time, connective tissue and the remnants of the bionanomaterial comprised, respectively, 46.6 ± 1.70% (95% CI 43.1–50.1%) and 9.7 ± 0.99 (95% CI 7.6–11.8%).

**Figure 10:**
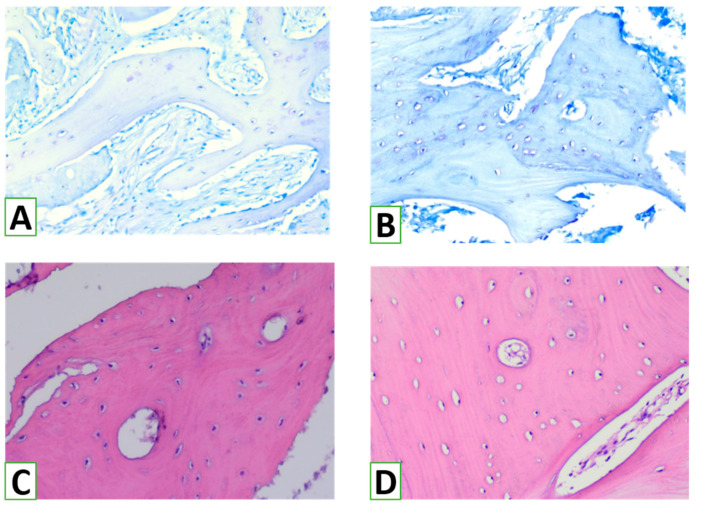
Heterogeneity of bone trabeculae structure and maturity within the zones of augmentation. Most of the bone trabeculae corresponded to a 2–3 score of osteogenesis. Remnants of HA/β-TCP were mostly resorbed and surrounded by primary bone (**A**), with irregular matrix formation and osteocyte distribution that was later replaced by secondary bone (**B**), with well-seen plates and regular orientation of osteocytes in the lacunae (**C**), with further osteocyte formation around channels with blood vessels (**D**). (**A**,**B**)—Toluidine blue staining, demonstrating newly formed bone trabeculae formed by primary bone, with further replacement by secondary bone. Magnification ×100. (**C**,**D**)—Hematoxylin and eosin staining, representing maturation of bone trabecules made by secondary bone with osteons. Magnification ×200.

Overall, osteogenesis was mostly graded as 3 (more than 40% of trabeculae) according to the scale (Table 2). Bone trabeculae of the 2nd and 4th scores were found in nearly 20–28%, and only a few areas represented initial phases of osteogenesis (Figure 10). Features of active remodeling were revealed in most cases. There were thick trabeculae demonstrating no HA/β-TCP remnants but features of replacement of the primary bone tissue by a secondary one. Primary bone tissue with random organization of matrix and lacunae containing osteocytes was surrounded by the lamellae of secondary bone tissue, with regularly organized light and dark bone lamellae.

**Figure 11:**
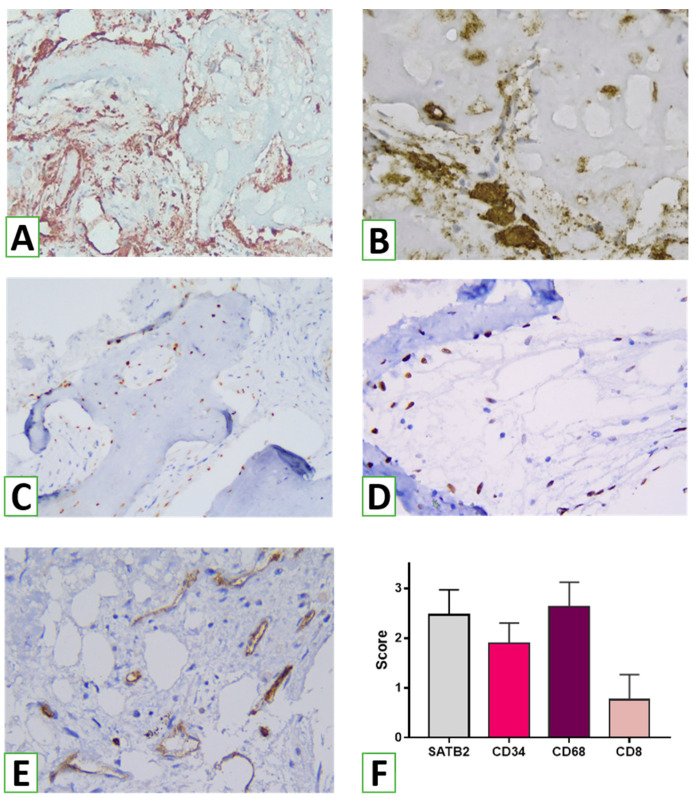
Cell types inside the zones of sinus augmentation. Numerous macrophages (**A**) and osteoclast macrophages (**B**) were found on the surface and between the bone trabeculae. Augmentation of sinuses by HA/β-TCP was associated with the differentiation and recruitment of osteogenic cells (**C**,**D**) found within bone tissue and in the connective tissue between trabeculae. Bone remodeling was also accompanied by angiogenesis (**E**). (**A**)—numerous CD163+ macrophages around and between trabeculae, IHC, magnification ×40; (**B**)—CD68+ osteoclasts on the surface of the resorbed trabeculae, IHC, magnification ×400; (**C**,**D**)—osteogenic cells (SATB2+) around and between newly formed trabeculae, IHC, magnification ×100 and ×400, respectively. (**E**)—CD34+ endothelial cells reflecting angiogenesis, IHC, magnification ×400. (**F**)—bar chart, demonstrating the semi-quantitative scores of different cell counts.

Remodeling features were associated with dense infiltration of the surrounding connective tissue by macrophages (with numerous CD163+ cells). Interestingly, abundant CD68+ cells (including macrophages and large osteoclasts) were distributed around the NG remnants and between trabeculae, while CD163+ macrophages were numerous, both near and around bone trabeculae (Figure 11). The architecture of the bone trabeculae intercalated with channels filled by connective tissues with numerous vessels. IHC revealed numerous CD34+ cells. In addition, various vessels were found around forming bone, demonstrating good blood supply. The surface of the network of bone trabeculae was covered by a layer of osteoblasts positive for SATB2. In addition, numerous recruited osteogenic cells were visualized around the bone trabeculae or in the perivascular areas. Overall, the assessment of various cell numbers demonstrated the prevalence of macrophages and osteogenic cells within the zones of augmentation (Figure 11F).

**Figure 12:**
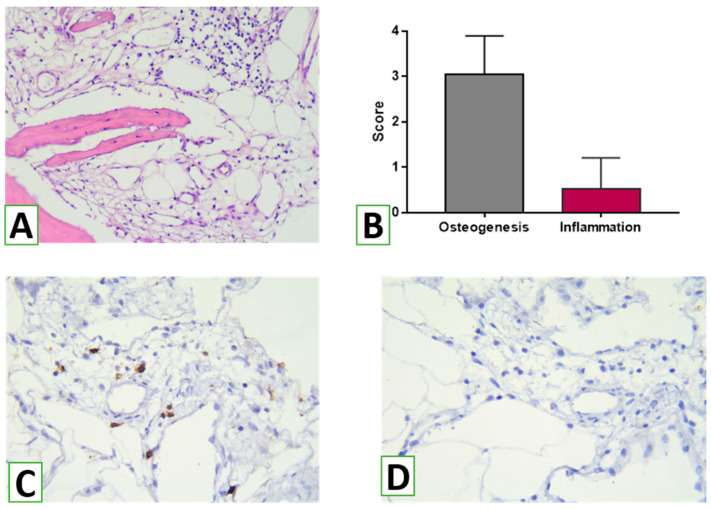
Mild inflammatory infiltration of the periodontal connective tissue at the place of augmentation (**A**) with few T cytotoxic cells (**B**) and a lack of Treg lymphocytes. (**A**)—hematoxylin and eosin staining. Magnification ×100. (**B**)—bar chart representing the scores of osteogenesis and immune reaction to HA/β-TCP at the zones of augmentation. (**C**,**D**)—immunohistochemistry using monoclonal antibodies to CD8 and FOXP3. Magnification ×400.

An assessment of immune infiltration and adaptive immune cell response demonstrated that HA/β-TCP was immune inert. Overall, the score for the inflammatory reaction was low (Figure 12). Inflammatory infiltration was judged as 0 or 1. Histological examination did not reveal features of acute or chronic inflammation within the observed samples. There was only slight local inflammatory infiltration in one case, with a mild accumulation of lymphocytes in the connective tissue out of the newly formed bone trabeculae. IHC revealed scarce CD8+ cells. At the same time, we did not find FOXP3+ cells in the observed specimens.

## 4. Discussion

The data obtained in the study indicate the effective use of HA/β-TCP nanomaterial with cells and tissues in alveolar processes. In most cases, the remnants of the biomaterial were determined within the bone trabeculae, and only a small portion was found extra-trabecularly, demonstrating high integration of the nanomaterial with new bone formation in the sinus lifting zone. It should be emphasized that the biomaterial used for sinus augmentation was immunologically inactive. According to histomorphometric analysis, a large area of the transplant residues in the augmentation zones was in contact with the newly formed bone tissue, reflecting the mechanisms of the osteoconductive effect of the novel HA/β-TCP bioactive nanomaterial. As previous studies have shown, the amount of newly formed bone, transplant residues, and connective tissue components varies widely when using different materials for sinus lifting [36]. For example, the use of autogenous bone stimulated osteogenesis to a greater extent compared to biphasic calcium phosphate (BCP) [37]. According to this study, 6–8 months after sinus lifting, the percentage of newly formed bone in the augmentation zone was 28.2% and 36.8% for BCP and autogenous bone, respectively. The majority of the areas of interest were occupied by connective tissue, forming 38.9% and 58.4%, respectively. In this case, fragments of BCP residues accounted for up to 32.9%, reflecting the limited biodegradation of the material. Instead, when using autogenous bone, fragments of its residues formed an average of 4.8% of the total volume of biopsies from the augmentation zones, which is the basis for positioning this material as the gold standard [38]. The results obtained in the study significantly exceed the indicators for BCP and approach the parameters when using autologous bone tissue, which actually reflects the high biocompatibility and, at the same time, appropriate biodegradation of novel HA/β-TCP, which acted as a conductor for the formation of its own bone tissue. The results demonstrated the advantages of using novel HA/β-TCP compared to allogeneic bone, which resulted in only 18.65 ± 12.20% new bone formation, 25.93 ± 12.36% residual allogeneic material, and 53.45 ± 10.34% connective tissue [39]. However, it should be noted that recent clinical studies on the use of combined scaffolds based on hydroxyapatite and polylactic acid or polyethyleneimine, which combine the characteristics of biodegradable polymers and bioceramics, have yielded results comparable to those presented in this study [40].

Histomorphometric analysis made it possible to evaluate not only the proportion and interaction between newly formed bone and the novel HA/β-TCP material’s residues but also to determine the degree of maturation of newly formed bone tissue in the area of transplant use. According to the data of the study, the majority of bone trabeculae in the biopsies of the augmentation zones corresponded to the third stage of osteogenesis—bone remodeling with the replacement of coarse fibrous bone tissue with lamellar bone. At the same time, signs of high maturity with the presence of osteons were found in more than a quarter of the trabeculae. The obtained data were compared with those in a study of five other bone materials [14]. The formation of bone and effective direct osteogenesis are actually a result of the activity of bone tissue cells—primarily osteoblasts and osteoclasts involved in the processes of osteogenesis and bone remodeling.

In this study, a significant number of SATB2-expressing osteogenic cells were identified in the augmentation zone. In addition to SATB2+ cells on the surface of bone trabeculae, a significant number of committed osteogenic cells were found freely in the connective tissue between trabeculae. These SATB2-positive cells may actually correspond to induced precursor cells that have been involved in the process of osteogenic differentiation. Such a pattern may reflect the osteoinductive potential of the HA/β-TCP nanomaterial, which stimulated the differentiation and migration of osteogenic cells to areas of osteogenesis.

According to the results of the histological and immunohistochemical studies, use of the novel HA/β-TCP nanomaterial was accompanied by signs of a slight inflammatory reaction. Within the biopsy specimens, only small diffuse lymphohistiocytic infiltrates were identified, which, according to various authors, may be a consequence of a transient weak immune response in response to damage in the augmentation zone and a normal bone remodeling process (Schmidt-Bleek K et al., 2012). Moreover, only a small number of CD8+ T cells were detected, which also play a role in osteogenesis and bone remodeling [41]. Similar data were obtained by Solakoglu Ö et al., who demonstrated the presence of a small number of infiltrates and the presence of CD3, CD4+, and CD8+ lymphocytes when using different variants of bone allografts.

It is important to emphasize that T regulatory cells responsible for the mechanisms of immune tolerance were not detected in the tissue biopsy samples from the augmentation zones during the study. The obtained results may indicate the primary low immunogenicity of the HA/β-TCP bone nanomaterial used. The role of other mechanisms of anti-inflammatory action associated with the activity of different subtypes of macrophages cannot be excluded either.

In addition to T lymphocytes, macrophages play an important role and have crucial significance for bone metabolism and bone tissue remodeling [22]. Macrophages represent a numerous population of immune cells present in different tissues and organs. Traditionally, macrophages quickly accumulate in damaged areas or areas of infection, where they play a critical role in innate immunity [42]. In addition, macrophages regulate tissue homeostasis and the implementation of various pathophysiological processes, including innate and adaptive immunity, regeneration, angiogenesis, and carcinogenesis. Moreover, macrophages not only initiate tissue inflammation but also promote tissue repair and remodeling [43]. In bone tissue, macrophages are an integral component of the bone remodeling process, as they coordinate the communication between osteoclasts and osteoblasts and stimulate anabolic processes critical for bone formation [44].

It is generally accepted that macrophages represent a spectrum of activated phenotypes rather than discrete stable subpopulations. Indeed, numerous studies have documented their programming flexibility, whereby macrophages switch from one functional phenotype to another in response to variable signals from the local microenvironment [45]. Schematically, macrophages are classified into two subsets: classically activated macrophages (M1) and alternatively activated macrophages (M2), although this is an oversimplification, and the actual spectrum of macrophage phenotypes is more complex [43].

As shown by the results of this study, numerous macrophages were detected in the biopsy samples from the augmentation zone, both CD68+ and CD163+. The dense network of CD163-positive M2 macrophages was of particular interest. Xia Z et al. (2006) previously showed that macrophages are the dominant cell type in the infiltration formed in response to the implantation of bionanomaterials in both soft and hard tissues. These cells and their variants, including multinucleated giant foreign body cells, are part of the inflammatory response and reaction to foreign material that occurs in any interventions involving biological materials. In addition, macrophages play an important role in the biodegradation of biomaterials used for implantation through the initiation of phagocytosis and extracellular degradation mechanisms.

## 5. Conclusions

In conclusion, the development and evaluation of the novel calcium deficient HA/β-TCP bone grafting nanomaterial presented in this study has demonstrated its potential for wide clinical application in maxillofacial surgery and general orthopedics practice. The two-step synthesis with freezing and calcination stages results in a highly porous nanomaterial with efficient osteoconductive properties, while the β-TCP phase provides the material with high biocompatibility, osteoinductive properties, and blood clotting ability. The HA phase balances mechanical properties and provides structural integrity throughout all stages of osteogenesis. The clinical trial has shown advanced bioinductive properties, inducing osteogenic cell recruitment, direct osteogenesis activation, and angiogenesis without a significant immune reaction. The unique combination of structural integrity, degradation properties, and bioactive response makes this bionanomaterial a promising candidate for a wide range of clinical applications in bone regeneration and augmentation. Further studies are necessary to explore its full potential and optimize its clinical application.

## Figures and Tables

**Table 1:** Characteristics of biomarkers used for immunohistochemical assessment of augmentation biopsies.

Biomarker	Description	Antibody Manufacturer	Clone
CD8	CD8 is an integral membrane glycoprotein found on the surface of cytotoxic T lymphocytes.	DAKO, Agilent	C8/144B
FOXP3	Forkhead Box P3 (FOXP3)is a transcription regulator, essential for the development and suppressive function of regulatory T cells (Treg).	Cell Marque,	EP340
CD68	CD68 antigen is a member of the lysosomal/endosomal-associated membrane glycoprotein family typical for human monocytes and macrophages. It is also expressed in bone osteoclasts (Ashley JW, 2011).	DAKO, Agilent	KP1
CD163	CD163 is a macrophage-associated scavenger receptor, typically expressed in alternatively activated M2 type macrophages.	Cell Marque	MRQ-26
CD34	CD34 is an adhesion molecule marking endothelial cells at the sites of active angiogenesis (Siemerink MJ, 2012).	DAKO, Agilent	QBEnd 10
SATB2	SATB2 is a protein binding to DNA that is involved in transcriptional regulation of gene expression during osteoblast differentiation. It is involved in pre-osteoblast proliferation as well (Dowrey T, 2019).	Cell Marque	EP281

**Table 2:** Characteristics of osteogenesis in biopsies of augmentation zones.

Score	Description	The Rate of Trabeculae Having the Corresponding Features
0	Freely situated biomaterial. No features of osteogenesis around the remnants of the biomaterial.	3.2%
1	There are osteoblasts and osteoid around or within the graft	4.9%
2	The primary (reticulofibrose) bone with osteoblasts and osteocytes surrounds or interferes with the remnants of the graft. There are features of initial remodeling.	21.3%
3	The bone trabeculae are formed by secondary (lamellar) bone with primary osteons, osteocytes, and vessels. The remnants of primary bone and biomaterial are present.	42.5%
4	There is mature secondary bone with conventional osteons.	27.1%

## Data Availability

No additional information is available for this paper.
